# Regioisomers of singly bridged calix[6]crown-6 and their heavy alkali metal complexes: a molecular baseball glove for caesium(I)

**DOI:** 10.1107/S2052252521010563

**Published:** 2021-11-03

**Authors:** Seulgi Kim, Jong Hwa Jung, Shim Sung Lee, In-Hyeok Park

**Affiliations:** a Department of Chemistry and Research Institute of Natural Science, Gyeongsang National University, Jinju 52828, Republic of Korea; b Graduate School of Analytical Science and Technology (GRAST), Chungnam National University, Daejeon 34134, Republic of Korea

**Keywords:** calix[6]crown, template effects, caesium(I) ions, molecular baseball glove, host–guest complexes

## Abstract

We report the formation of heavy alkali metal complexes of bicyclic host molecules including the caesium(I) complex that catches the central metal ion with the deep pocket of the host similar to a baseball glove. For this, three regioisomers of singly bridged calix[6]crown-6 [1,2-bridged (H_4_ *L* ^1,2^), 1,3-bridged (H_4_ *L* ^1,3^) and 1,4-bridged (H_4_ *L* ^1,4^)] have been synthesized by alkyl­ation of calix[6]arene with penta­ethyl­ene glycol di­tosyl­ate in the presence of *M*_2_CO_3_ (*M* = Na, K, Rb and Cs). The larger the cation size of the metal carbonate, the higher the yield of the H_4_ *L* ^1,4^ isomer, indicating the size-based template effect. A combination of H_4_ *L* ^1,2^ and RbOH allowed isolation of the mononuclear rubidium(I) complex (**1**) in which the metal center is six-coordinated in a loose fashion, the remaining two oxygen donors in the crown loop and two phenols in the calix rim are uncoordinated. Notably, the complexation of H_4_ *L* ^1,2^ with CsOH yielded the mononuclear caesium(I) complex (**2**), in which all possible ten binding sites on the deep and good-fit pocket participate in coordination via high cooperativity between the crown loop and calix rim, similar to a baseball glove. In dipolar organic solution, the caesium(I) complex **2** remains intact. H_4_ *L* ^1,4^ afforded a dicesium(I) complex (**3**) and adjacent complexes are linked by intermolecular cation–π interactions, giving rise to a pseudo one-dimensional coordination polymer. These results provide insight into the metal carbonate-dependent synthesis of calix[6]crowns and the influence of regioisomers on caesium(I) complexation.

## Abstract 2

The syntheses of regioisomers of calix[6]crown-6 under a metal carbonate template were highly metal cation-dependent and the 1,2-bridged isomer (H_4_ *L* ^1,2^) catches Cs^+^ very efficiently in its deep and good-fit pocket similar to a baseball glove.

## 1. Introduction

Since the pioneering works of Pedersen, Lehn and Cram, supramolecular chemistry of macrocycles is still significant in chemistry and biology because many of the roots and concepts came from the phenomena found in biological process such as molecular recognition, self-assembly, enzyme catalysis and ion transport (Pedersen, 1967 ▸; Izatt *et al.*, 1995 ▸; Lindoy, 1990 ▸; Lehn, 1995 ▸). Supramolecular chemistry has been extended to various research areas such as metal–organic frameworks, metal–organic coordination cages, hydrogen-bonded organic frameworks, covalent organic frameworks, π–π stacked frameworks, as well as applications of macrocyclic complexes and continuous studies being carried out (Zhou *et al.*, 2012 ▸; Fujita *et al.*, 2005 ▸; Cook *et al.*, 2013 ▸; Chakraborty *et al.*, 2021 ▸; Deng *et al.*, 2020 ▸; Ouyang *et al.*, 2017 ▸, 2018 ▸; Li *et al.*, 2014 ▸, 2021 ▸; Park *et al.*, 2020 ▸; Britton *et al.*, 2021 ▸; Boer *et al.*, 2019 ▸; Braunecker *et al.*, 2018 ▸; Hong *et al.*, 2021 ▸; Yadava *et al.*, 2020 ▸). The advances of experimental instruments for structural analyses including single-crystal X-ray diffraction and high-field NMR have also led such progress.

Before the discovery of crown ethers by Pederson, ionophores for alkali metal ions were very rare except in some specified natural products, *e.g.* valinomycin and nonactin (Brockmann & Schmidt-Kastner, 1955 ▸; Brockmann & Geeren, 1957 ▸; Prestegard & Chan, 1969 ▸, 1970 ▸). So far, complexes of heavy alkali metal ions including Rb^+^ and Cs^+^ are relatively less explored than their lighter homologues such as Li^+^, Na^+^ and K^+^ (Izatt *et al.*, 1995 ▸; Gokel *et al.*, 2004 ▸). For the complexation of heavy alkali metal ions, hetero-di and polytopic receptors incorporating two different macrocycle units could be more advantageous due to the deep pocket being formed rather than simple flat macrocycles (Lehn, 1978 ▸). In this sense, calix[*n*]crowns are one of the most important host molecules (Asfari *et al.*, 2001 ▸; Harrowfield & Koutsantonis, 2007 ▸; Ikeda & Shinkai, 1997 ▸; Kim, Lee *et al.*, 2012 ▸; Nimse & Kim, 2013 ▸). Practically, some large crown ethers (Talanov *et al.*, 2000 ▸; Shinkai *et al.*, 1982 ▸; Rofouei *et al.*, 2010 ▸) and calix[4]crown derivatives (Ji *et al.*, 1999 ▸, 2001 ▸; Boda & Sheikh, 2012 ▸; Kim *et al.*, 2004 ▸, 2010 ▸; Choi *et al.*, 2006 ▸; Lee *et al.*, 2008 ▸; Kim, Lynch *et al.*, 2012 ▸; Bu *et al.*, 2004 ▸) have been utilized in complexation not only for heavy alkali metal ions but also for their radioactive elements including ^137^Cs^+^ (Russell *et al.*, 2014 ▸).

Considering the large size of Cs^+^ in the alkali metal family and its hard-acid nature, bridging of calix[6]arene with larger crown loops could be promising rather than those with calix[4]arene. Furthermore, this approach serves regioisomers depending on the bridging positions, some of which can possess a semi-flexible and deeper binding pocket. Shinkai *et al.* (1982 ▸) proposed the *O*-methyl­ation processes of *p-tert*-butyl­calix[6]arene in the presence of K_2_CO_3_ (Otsuka *et al.*, 1994 ▸). The Chen group reported the regioselective synthesis of 1,2-bridged *p-tert*-butyl­calix[6]-dioxocrowns in the presence of K_2_CO_3_ (Yang & Chen, 2001 ▸). Unlike the big advances in calix[4]crowns, the corresponding calix[6]crowns especially with larger crown loops have been relatively less explored because their functionalization by the selective bridging of two phenolic units could be complicated due to the greater number of sites (six OH groups) being attached (Gutsche, 1989 ▸; Blanda *et al.*, 2000 ▸; Chen & Li, 2001 ▸; Li *et al.*, 1999 ▸). The difficulty also arises from the isolation and characterization of pure regioisomers.

In this work, we accomplished regioselective syntheses of calix[6]crown-6 isomers (Scheme 1). Reactions of calix[6]arene with a di-electrophile can lead to the formation of three singly bridged regioisomers; 1,2-bridged (H_4_ *L* ^1,2^), 1,3-bridged (H_4_ *L* ^1,3^) and 1,4-bridged (H_4_ *L* ^1,4^). The regioisomer distribution in the product could be dependent on the reaction conditions including the base (Otsuka *et al.*, 1994 ▸). Thus, single-bridging reactions were accomplished between calix[6]arene and penta­ethyl­ene glycol di­tosyl­ate in the presence of different metal (Na, K, Rb and Cs) carbonates in xylene. 
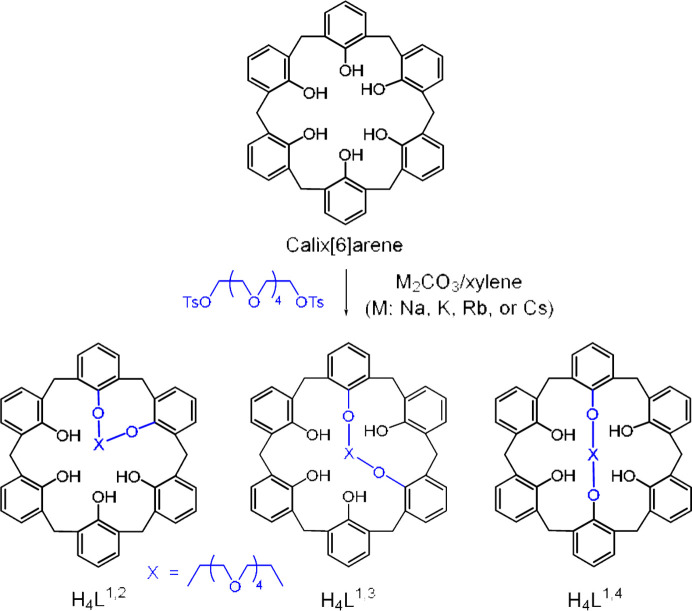


In addition to the synthesis for the above regioisomers, we focused our attention to the preparation and structural studies of their heavy alkali metal complexes including Cs^+^. Notably, the semi-flexible calix[6]crown regioisomers are expected to provide pockets with different depths and dimensions depending on the bridging positions. For example, 1,2-bridging makes a deeper pocket but 1,4-bridging produces a shallow one (Scheme 1). Furthermore, the phenol groups under the basic reaction conditions could be advantageous for a few reasons: (i) charge neutrality of the complexes can be achieved by phenolate groups, (ii) the phenolate group can acts as a strong cation-ligation site. In this regard, such modification of calix[6]arene entity by bridging with proper crown loops could be a potential design tool for engineering new coordination products in terms of stoichiometries, topologies and coordination modes. In our results, the metal carbonate template-based synthesis was highly cation-dependent. Here it is highlighted that one of the complexes isolated exhibits binding of Cs^+^ in the deep pocket of the host molecule similar to a baseball glove. The details are discussed below.

## 2. Results and discussion

### 2.1. Synthesis of three regioisomers of calix[6]crown-6

Calix[6]arene was prepared as reported previously (Casnati *et al.*, 1995 ▸). As shown in Scheme 1, regioisomers of the singly bridged calix[6]crown-6 were synthesized via direct alkyl­ation of calix[6]arene with penta­ethyl­ene glycol di­tosyl­ate in the presence of *M*_2_CO_3_ (*M* = Na, K, Rb and Cs). Depending on the metal carbonates used the regioisomer products were obtained in low to reasonable yields. The selectivity ratios were determined from the NMR data of the reaction mixtures as listed in Table 1 ▸. The same reactions in the presence of Na_2_CO_3_, K_2_CO_3_ or Rb_2_CO_3_ gave the selectivity ratio (%) of three regioisomers (H_4_ *L* ^1,2^:H_4_ *L* ^1,3^:H_4_ *L* ^1,4^): 75:15:0, 49:43:8 and 17:22:61. As the cation size becomes smaller, the 1,2-bridging is predominant. Moreover, when Cs_2_CO_3_ was used, only 1,3- and 1,4-bridging occurred with a 17:83 ratio, indicating the favorable 1,4-bridging by the largest cation, Cs^+^. On the basis of these results, we can explain that the cation size for the bases plays a crucial role as a template in the synthesis of the regioisomers. Some similar results on the bridging of *p-tert*-calix[6]arene with shorter polyethyl­ene glycol di­tosyl­ates have been reported by the Chen group (Li *et al.*, 1999 ▸).

### 2.2. Separation and identification of the regioisomers

Medium-pressure liquid column chromatography of the reaction mixture (ethyl acetate/*n*-hexane) on silica gel afforded 1,2-, 1,3- and 1,4-bridged isomers in pure form. The structural characterization of three isomers isolated was confirmed by ^1^H and ^13^C NMR (Figs. S1 and S2 of the supporting information). In the ^1^H NMR spectra, the conformations of H_4_ *L* ^1,2^, H_4_ *L* ^1,3^ and H_4_ *L* ^1,4^ could not be fully deduced owing to the complicated and overlapped peaks for O—C*H*_2_, Ar—C*H*_2_—Ar and Ar—*H*. Instead, the peak patterns of the four hydroxyl groups (Ar—O*H*) in each isomer serve as sensitive probes to distinguish the 1,2-, 1,3- or 1,4-bridging due to the different molecular symmetry levels (Table 2 ▸) (Cunsolo *et al.*, 1998 ▸; Geraci *et al.*, 1996 ▸).

In the spectrum for H_4_ *L* ^1,2^, the peaks for two non-equivalent Ar—O*H* groups on the 3- and 4-positions appear at 8.31 p.p.m. (2H) and 8.79 p.p.m. (2H), respectively [Table 2 ▸ and Fig. S1(*a*)]. Two singlets at 8.26 p.p.m. (1H) and 7.72 p.p.m. (3H) in the spectrum for H_4_ *L* ^1,3^ indicate one hydroxyl proton on the 2-position and three hydroxyl protons on the 3-, 4- and 5-positions in accidental equivalence, respectively [Table 2 ▸ and Fig. S1(*b*)]. One singlet at 7.75 p.p.m. (4H) in the spectrum for H_4_ *L* ^1,4^ is attributed to the four hydroxyl protons in equivalent positions due to the high symmetry [Table 2 ▸ and Fig. S1(*c*)]. In the ^13^C NMR spectra, as expected, the numbers of peaks for the 1,4-bridged isomer is much smaller than those of the 1,2- or 1,3-bridged isomers (Fig. S2).

### 2.3. Preparation of rubidium(I) and caesium(I) complexes of H_4_ *L* ^1,2^ (**1** and **2**)

**Figure 1:**
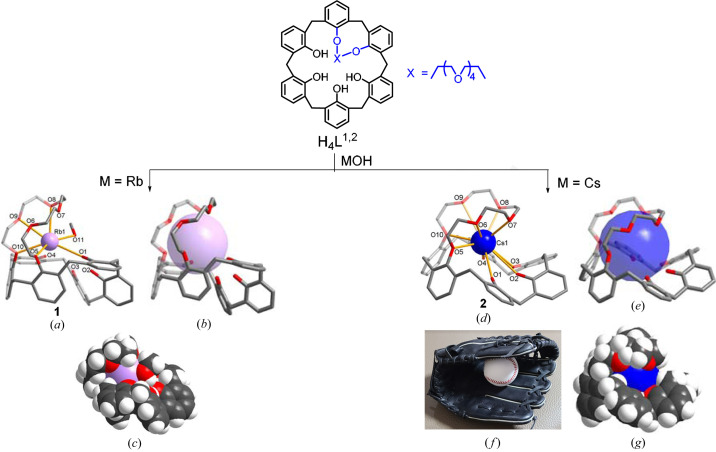
(*a*) Formation of the rubidium(I) complex of H_4_ *L* ^1,2^, [Rb(H_3_ *L* ^1,2^)(CH_3_OH)] (**1**) showing a six-coordinated environment; (*b*) loose capture of Rb^+^; and (*c*) space-filling structure. (*d*) Formation of the caesium(I) complex of H_4_ *L* ^1,2^, [Cs(H_3_ *L* ^1,2^)]·3CHCl_3_ (**2**) showing a ten-coordinated environment; (*e*) tight capture of Cs^+^ in the deep pocket similar to (*f*) a baseball glove; and (*g*) space-filling structure. Non-coordinated solvent molecules are not shown.

As mentioned, the use of alkali metal hydroxides in the complexation study was successful in isolating solid products. First, the reaction of H_4_ *L* ^1,2^ with RbOH in chloro­form/methanol afforded a colorless crystalline product (**1**) in low yield (5%). When the same synthetic procedure was repeated with CsOH, a colorless crystalline product (**2**) was obtained in a much higher yield (70%). X-ray analysis revealed that both products feature mononuclear complexes but their coordination environments are different (Fig. 1 ▸).

The rubidium(I) complex **1** crystallizes in the monoclinic space group *P*2_1_/*n* (Tables S1 and S2 of the supporting information). The structure features a 1:1 (metal-to-ligand) complex of type [Rb(H_3_ *L* ^1,2^)(CH_3_OH)] [Figs. 1 ▸(*a*)–(*c*)]. The rubidium(I) center is six-coordinated by four ether oxygens [Rb1—O 2.911 (17)–3.143 (2) Å], one phenolate oxygen [Rb1—O1 3.080 (18) Å] and one methanol molecule [Rb1—O11 2.879 (3) Å] [Fig. 1 ▸(*a*)]. The calix rim shows a bent cone shape mainly due to two bridging phenol units on 1,2-positions and two phenol units on 4,5-positions showing an up conformation with the flattened conformation of two remaining phenol units on the 3,6-positions. Two ether oxygens (O6 and O7) and three phenol groups remain uncoordinated, indicating that the semi-rigid pocket is somewhat larger than the cation, creating a loose structure [Figs. 1 ▸(*b*) and 1(*c*)]. Although the synthesis of **1** is reproducible, the yield is quite low (5%) probably due to low stability.

The caesium(I) complex **2** crystallizes in the monoclinic space group *P*2_1_/*n* (Tables S1 and S3). Again, the structure features a 1:1 (*meta*-to-ligand) complex of type [Cs(H_3_ *L* ^1,2^)]·3CHCl_3_ [Figs. 1 ▸(*d*)–1(*g*)]. The caesium(I) center is *fac*-coordinated by six ether oxygens [Cs1—O 3.056 (2)–3.553 (7) Å] [Fig. 1 ▸(*d*)]. The remaining sites are occupied by four phenol oxygens to yield an overall metal coordination of ten. As we understand, this is an example in which Cs^+^ possesses the maximum coordination number (CN = 10). Accordingly, the caesium(I) in **2** is tightly captured by the bicyclic pocket in a baseball glove like manner [Figs. 1 ▸(*e*)–(*f*)]. Among the four bonds between Cs1 and phenol oxygens, the Cs1—O4 [3.187 (2) Å] is much shorter than the other three bonds [3.377 (2)–3.653 (2) Å], indicating that the O4 atom is deprotonated [Fig. S3(*b*)]. Unlike that in the rubidium(I) complex **1**, the 1,2-bridged isomer in **2** effectively shields the caesium(I) center from the solvent molecules [Figs. 1 ▸(*b*)–1(*d*)].

In **2**, the crown loop is somewhat ellipsoidal, mainly due to the 1,2-bridging with a narrow distance and all the ether oxygens are associated with a *gauche* arrangement [torsion angles of O—(CH_2_)_2_—O 35.0 (4)–78.5 (9)°] adopting a shrunken and folded conformation [Fig. 1 ▸(*d*) and 1(*e*)]. In this case, the calix rim exhibits a partial flattened cone shape due to four unsubstituted phenolates on the 3,4,5,6-positions showing a flattened conformation and two bridging phenol units on 1,2-positions with an up conformation. Considering that synthetic hosts for heavy alkali metal ions usually employ 6 to 8 binding interactions, the observed 10 bindings in **2** represents a good example of the highest coordination number in the deep and good-fit pocket, reflecting the efficient inclusion of caesium(I) to achieve maximum stabilization. Consequently, the preferred formation of **2** results in the three ligating components (crown loop, phenol and phenolate) toward caesium(I) being optimally fitted to satisfy the geometric and electronic requirements that appear to be a baseball glove structure.

### 2.4. Preparation of the dicesium(I) complex of H_4_L^1,4^ (**3**)

**Figure 2:**
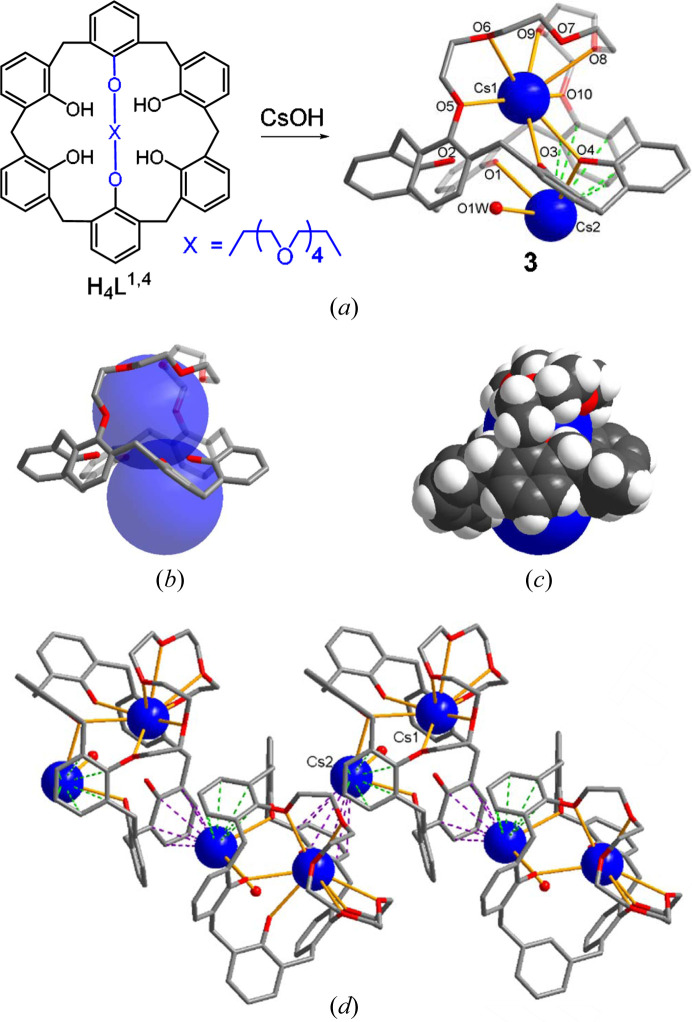
(*a*) Formation of the dicesium(I) complex of H_2_ *L* ^1,4^, {[Cs_2_(H_2_ *L* ^1,4^)(H_2_O)]·CHCl_3_}*_n_* (**3**); (*b*) capture of two Cs^+^; (*c*) space-filling structure; and (*d*) pseudo one-dimensional polymeric zigzag chain via intermolecular cation–π interactions (purple dashed lines).

When H_4_ *L* ^1,4^ was reacted with CsOH under identical conditions, colorless crystalline **3** was isolated. X-ray analysis revealed that **3** crystallizes in the monoclinic space group *P*2_1_/*c* (Tables S1 and S4). Unlike **2**, this product features a dinuclear complex with the formula {[Cs_2_(H_2_ *L* ^1,4^)(H_2_O)]·CHCl_3_}*_n_* (Fig. 2 ▸). In **3**, two Cs^+^ ions (Cs1 and Cs2) show very different coordination environments [Fig. 2 ▸(*a*)]. For example, the Cs1 atom mainly occupies the cavity center of the crown loop because the 1,4-bridging in **3** provides a wider and shallow pocket than the 1,2-bridging does in **2** [Fig. 2 ▸(*b*)]. In fact, the distance between two bridging oxygens in **3** [O5⋯O10 6.138 (4) Å] is almost twice of that in **2** [3.109 (3) Å]. Thus, the H_4_ *L* ^1,4^ could have some extra empty space inside the calix rim where the second Cs^+^ (Cs2 atom) is located [Fig. 2 ▸(*c*)]. The Cs1⋯Cs2 separation (5.014 Å) is slightly longer than twice of the van der Waals radius of Cs^+^ (2.35 Å). Diverse types of dicesium(I) compounds formed by inorganic counter ions are known (Kříž *et al.*, 2011 ▸), but the corresponding dicesium(I) complexes stabilized by organic ligands are relatively rare (Duraisamy *et al.*, 2020 ▸).

In **3**, the Cs1 atom is seven-coordinated, with five coordination sites occupied by crown ether oxygens [Cs1—O 3.098 (3)–3.376 (4) Å, average 3.201 (5) Å] and the remaining sites occupied by two phenol oxygens [Cs1—O3 3.145 (3), Cs1—O4 3.200 (3) Å]. Two phenol groups (O1 and O4) are deprotonated [Fig. S3(*c*)]. Again, all the ether oxygens on the crown loop are associated with the *gauche* conformation [torsion angles of O—(CH_2_)_2_—O 54.1 (5)–64.3 (6)°]. However, the Cs2 atom is three-coordinated being bound by two phenol oxygens [Cs2—O1 3.046 (3), Cs2—O4 3.063 (3) Å] and one water molecule. In addition, the Cs2 atom shows two different η^6^-type cation⋯π interactions from one ligand [green dashed lines, Cs2⋯C 3.420 (5)–3.825 (4) Å, average 3.633 (10) Å] and another ligand [purple dashed lines, Cs2⋯C 3.489 (5)–3.974 (4) Å, average 3.726 (11) Å] resulting in the formation of a pseudo one-dimensional zigzag polymer structure as a first example of this type [Fig. 2 ▸(*d*)]. Recently, our group reported a poly(sandwich)-type caesium(I)complex of bis-*o*-xylyl-(17-crown-5) (Kim *et al.*, 2020 ▸).

Consequently, the 1,4-bridging provides a wider and shallow pocket which occupies two Cs^+^ ions. In stabilizing the Cs1 atom, the crown loop acts as the primary binding sites, in addition to some phenol groups. The Cs2 atom is mainly stabilized by the calix rim unit, indicating the partial cooperativity between the crown loop and calix rim. Attempts to isolate some other heavy alkali metal complexes of H_4_ *L* ^1,3^ and H_4_ *L* ^1,4^ were not successful.

### 2.5. NMR studies of the caesium(I) complexes

**Figure 3:**
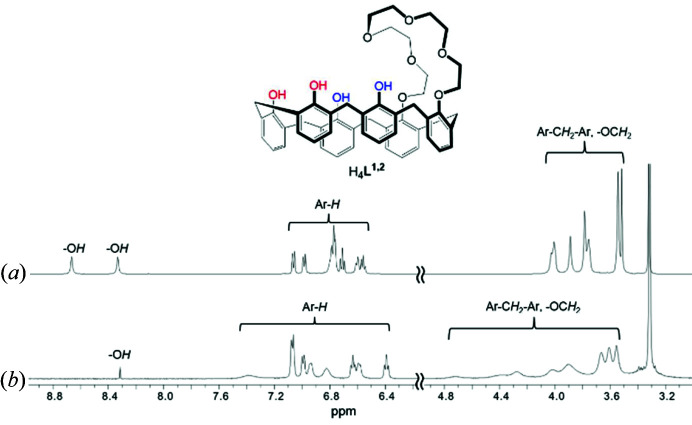
^1^H NMR spectra of (*a*) H_4_ *L* ^1,2^ and (*b*) [Cs(H_3_ *L* ^1,2^)]·3CHCl_3_ (**2**) in DMSO-*d*_6_.

**Figure 4:**
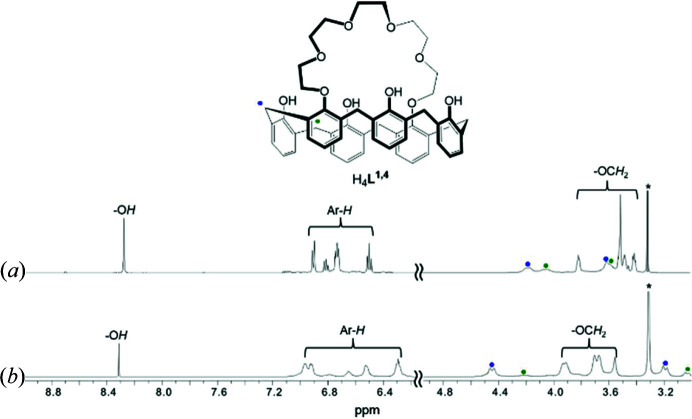
^1^H NMR spectra of (*a*) H_4_ *L* ^1,4^ and (*b*) {[Cs_2_(H_2_ *L* ^1,4^)(H_2_O)]·CHCl_3_}*_n_* (**2**) in DMSO-*d*_6_.

The ^1^H-NMR spectra of the caesium(I) complexes **2** and **3** in DMSO-*d*_6_ were observed and compared with their free forms (Figs. 3 ▸ and 4 ▸). Compared with the spectrum of free H_4_ *L* ^1,2^, its caesium(I) complex **2** shows remarkable peak broadening, with the signal of OCH_2_ of the crown loop being shifted downfield because of the presence of multiple Cs—O bonds restricting the conformational mobility of the ligand (Fig. 3 ▸). This observation illustrates that not only the six ether oxygens of the crown loop but also the four phenol oxygens are strongly involved in the interaction with Cs^+^, indicating that the baseball glove structure remains intact. The similar line broadening and Cs^+^-induced low-field shifts for **3** were also observed but the magnitudes are smaller, probably the 1,4-isomer interacts weakly with Cs^+^ than the 1,2-isomer does (Fig. 4 ▸). Consequently, it is obvious that the inclusion of Cs^+^ is highly regulated by the bridging positions and the products including the baseball glove-type species are stable in the dipolar aprotic medium. Due to the solubility problem, we were not able to obtain the binding constants for the complexations.

## 3. Conclusions

With the aim of developing a novel host system for heavy alkali metal ions including Cs^+^, our syntheses of three regioisomers (H_4_ *L* ^1,2^, H_4_ *L* ^1,3^ and H_4_ *L* ^1,4^) in the presence of *M*_2_CO_3_ (*M* = Na, K, Rb and Cs) exhibited the size-based template effect. In the complexations with heavy alkali metal hydroxides, we probed the factors influencing the preferential formation of a baseball glove-type caesium(I) complex by employing the 1,2-bridging isomer (H_4_ *L* ^1,2^). Our NMR study also supports that such caesium(I) complex is stable in solution. Consequently, the optimized baseball glove-type complexation is strongly associated with the 1,2-bridging of calix[6]crown-6, which provides the deeper and good-fit pocket for Cs^+^ in comparison with other bridging types.

## Supplementary Material

Crystal structure: contains datablock(s) 1, 2, 3. DOI: 10.1107/S2052252521010563/yc5035sup1.cif

Structure factors: contains datablock(s) 1. DOI: 10.1107/S2052252521010563/yc50351sup2.hkl

Structure factors: contains datablock(s) 2. DOI: 10.1107/S2052252521010563/yc50352sup3.hkl

Structure factors: contains datablock(s) 3. DOI: 10.1107/S2052252521010563/yc50353sup4.hkl

Supporting figures and tables. DOI: 10.1107/S2052252521010563/yc5035sup5.pdf

CCDC references: 2084082, 874813, 874814

## Figures and Tables

**Table 1:** Base-dependent synthesis of three regioisomers of singly bridged calix[6]crown-6 Reaction conditions: calix[6]arene (1.0 equiv), base (2.0 equiv), penta­ethyl­ene glycol di­tosyl­ate (0.9 equiv), xylene (500 ml), reflux. Selectivity ratios were determined by ^1^H NMR.

	Selectivity ratio (%)			
Base	H_4_ *L* ^1,2^	H_4_ *L* ^1,3^	H_4_ *L* ^1,4^	Total yield (%)
Na_2_CO_3_	75	15	10	32.4
K_2_CO_3_	49	43	8	33.6
Rb_2_CO_3_	17	22	61	32.5
Cs_2_CO_3_	0	17	83	35.9

**Table 2:** ^1^H NMR resonances for the phenolic protons of three regioisomers in CDCl_3_

	H_4_ *L* ^1,2^	H_4_ *L* ^1,3^	H_4_ *L* ^1,4^
Ar—O*H* (δ, p.p.m.)	8.31 (2H) 8.79 (2H)	8.26 (1H) 7.72 (1H) 7.72 (2H)	7.75 (4H)
