# Supplementary material for: Regioisomers of singly bridged calix[6]crown-6 and their heavy alkali metal complexes: a molecular baseball glove for caesium(I)
Source: IUCrJ. 2021 Nov 3;9(Pt 1):43–8. doi: 10.1107/S2052252521010563 (PMC8733876; doi:10.1107/S2052252521010563)
Supplement: Supplementary file 5 [file m-09-00043-sup5.pdf]

IUCrJ

Volume 9 (2022)

Supporting information for article:

Regioisomers of singly bridged calix[6]crown-6 and their heavy alkali metal complexes: A molecular baseball glove for cesium(I)

Seulgi Kim, Jong Hwa Jung, Shim Sung Lee and In-Hyeok Park

Experimental Section

General. All chemicals and solvents used in the synthetic works were of reagent grade and were used without further purification. The FT-IR spectra were measured with a Nicolet *i*S10 spectrometer. Each product obtained in this work was dried in a vacuum before elemental analysis, which was carried out on a Thermo Scientific Flash 2000 Series elemental analyzer. The nuclear magnetic resonance (NMR) spectra were recorded on a Bruker 500 spectrometers (500 MHz). The electrospray ionization (ESI) mass spectra were obtained on a Thermo Scientific LCQ Fleet spectrometer. The fast atom bombardment (FAB) ionization mass spectra were obtained on a JMS-700 MSTATION spectrometer.

Syntheses of Regioisomers of Calix[6]crown-6 ($H_4L^{1,2}$, $H_4L^{1,3}$, and $H_4L^{1,4}$). A mixture of calix[6]arene (5.01 g, 7.87 mmol), pentaethylene glycol ditosylate (3.88 g, 7.08 mmol), and anhydrous K_2CO_3 (2.17 g, 15.7 mmol) in xylene (500 mL) was stirred at reflux temperature for 24 h. After removal of solvent under reduced pressure, the residue was treated with HCl (10%, v/v) and extracted with $CHCl_3$. The organic layer was separated, dried over Na_2SO_4, filtered and concentrated. The resulting solid was later shown to contain a mixture of $H_4L^{1,2}$ (49%), $H_4L^{1,3}$ (43%) and $H_4L^{1,4}$ (8%). This mixture was separated by column chromatography on silica gel (eluent: hexane/ethyl acetate = 1:2) (yield 37%). Three regioisomers were obtained as a pale- yellow solid.

1,2-Bridged Calix[6]crown-6 ($H_4L^{1,2}$). Mp: 154-156 °C. Anal. Calcd for $C_{52}H_{54}O_{10}$: C, 74.44; H, 6.49. Found: C, 74.61; H, 6.48. IR (KBr pellet): 2871, 1593, 1466, 1355, 1300, 1249, 1214, 1123, 1085, 1048, 946, 834, 755 cm^{-1}. MS (ESI) *m/z*: 861.50 $[(H_4L^{1,2})Na]^+$. ^{1}H NMR (500 MHz, $CDCl_3$): (See Figure 1a for the assignment of each proton) δ 8.79 (*s*, 2H, ArO*H*), 8.31 (*s*, 2H, ArO*H*), 7.25-6.78 (*overlapped*, 18H, Ar*H*) 4.14-3.71 (*overlapped*, 32H, ArCH_2Ar and OCH_2CH_2). ^{13}C NMR (125 MHz, $CDCl_3$): (See Figure 2a for the assignment of each carbon) δ 153.3, 151.3, 150.2, 134.04, 133.98, 129.4, 129.3, 129.0, 128.8, 128.6, 128.1, 128.0, 127.9, 127.8, 127.7, 125.7, 121.3, 120.7, 73.1, 71.2, 70.8, 70.3, 31.7, 31.6, 31.1 ,31.0.

1,3-Bridged Calix[6]crown-6 ($H_4L^{1,3}$). Mp: 145-147 °C. Anal. Calcd for $C_{52}H_{54}O_{10}$: C, 74.44; H, 6.49. Found: C, 74.18; H, 6.44. MS (ESI) *m/z*: 861.58 $[(H_4L^{1,3})Na]^+$. ^{1}H NMR (500 MHz, $CDCl_3$): (See Figure 1b for the assignment of each proton) δ 8.26 (*s*, 1H, ArO*H*), 7.72 (*s*, 3H, ArO*H*), 7.13-6.70 (*overlapped*, 18H, Ar*H*) 4.03-3.50 (*overlapped*, 32H, ArCH_2Ar and OCH_2CH_2). ^{13}C NMR (125 MHz, $CDCl_3$): (See Figure 2b for the assignment of each carbon) δ 153.5, 152.0, 151.3, 150.9, 133.8, 133.3, 129.6, 129.2, 129.1, 129.0, 128.8, 127.9, 127.81, 127.76, 127.2, 127.1, 124.8, 121.0, 120.8, 119.9, 73.1, 71.2, 70.7, 70.6, 70.2, 31.7, 31.3, 30.9.

1,4-Bridged Calix[6]crown-6 ($H_4L^{1,4}$). Mp: 145-147 °C. Anal. Calcd for $C_{52}H_{54}O_{10}$: C, 74.44; H, 6.49. Found: C, 74.03; H, 6.39. IR (KBr pellet): 2919, 1458, 1354, 1269, 1197, 1086, 942, 836, 753 cm^{-1}. MS (ESI) *m/z*: 861.58 $[(H_4L^{1,4})Na]^+$. ^{1}H NMR (500 MHz, $CDCl_3$): (See Figure 1c for the assignment of each proton) δ 7.75 (*s*, 4H, ArO*H*), 7.12-6.75 (*overlapped*, 18H, Ar*H*) 4.43-3.55 (*overlapped*, 32H,

Ar*CH$_2$*Ar and O*CH$_2$CH$_2$*). ^{13}C NMR (125 MHz, $CDCl_3$): (See Figure 2c for the assignment of each carbon) *δ* 152.6, 151.8, 133.4, 129.1, 128.8, 128.7, 1127.7, 127.6, 125.4, 120.3, 74.8, 71.3, 71.0, 70.0, 31.6, 31.2.

Preparation of [Rb($H_3L^{1,2}$)(CH_3OH)] (1): Rubidium hydroxide (21.6 mg, 0.144 mmol) in methanol (3 mL) was added into a chloroform solution (3 mL) of $H_4L^{1,2}$ (20.1 mg, 0.0240 mmol) at room temperature. Slow evaporation of the reaction mixture at room temperature afforded a colorless solid product (yield 5%). Mp: 212-213 °C (decomp.). Anal. Calcd for $C_{54}H_{58}Cl_3RbO_{11}$: C, 60.34; H, 5.44. Found: C, 60.62; H, 5.58. IR (KBr pellet): 3408, 2913, 1630, 1589, 1448, 1354, 1192, 1083, 1052, 943, 751 cm^{-1}. MS (FAB) *m/z*: 923 [($H_4L^{1,2}$)Rb]$^+$.

Preparation of [Cs($H_3L^{1,2}$)]·$CHCl_3$ (2): Cesium hydroxide (21.6 mg, 0.144 mmol) in methanol (3 mL) was added into a chloroform solution (3 mL) of $H_4L^{1,2}$ (20.1 mg, 0.0240 mmol) at room temperature. Slow evaporation of the reaction mixture at room temperature afforded a colorless solid product (yield 70%). Mp: 243-246 °C (decomp.). Anal. Calcd for $C_{53}H_{54}Cl_3CsO_{10}$: C, 58.39; H, 4.99. Found: C, 57.96; H, 4.91. IR (KBr pellet): 2870, 1592, 1466, 1450, 1351, 1301, 1249, 1218, 1085, 1045, 923, 835, 755 cm^{-1}. MS (FAB) *m/z*: 971.33 [($H_4L^{1,2}$)Cs]$^+$.

Preparation of {[Cs_2($H_2L^{1,4}$)(H_2O)$_2$]·2$CHCl_3$}$_n$ (3): Cesium hydroxide (21.6 mg, 0.144 mmol) in methanol (3 mL) was added into a chloroform solution (3 mL) of $H_4L^{1,4}$ (20.3 mg, 0.0242 mmol) at room temperature. Slow evaporation of the reaction mixture at room temperature afforded a colorless solid product (yield 55%). Mp: 260-263 °C (decomp.). Anal. Calcd for $C_{54}H_{58}Cl_6Cs_2O_{12}$: C, 47.08; H, 4.24. Found: C, 46.96; H, 4.17. IR (KBr pellet): 3421, 2903, 2360, 1594, 1459, 1350, 1289, 1250, 1085, 949, 841, 754 cm^{-1}. MS (FAB) *m/z*: 1103.25 [($H_3L^{1,4}$)Cs_2]$^+$.

X-ray Crystallographic Analysis

All data were collected on a Bruker SMART APEX II ULTRA diffractometer equipped with graphite monochromated Mo K_α radiation (λ = 0.71073 Å) generated by a rotating anode. Data collection, data reduction, and semi-empirical absorption correction were carried out using the software package of APEX2.[S1] All of the calculations for the structure determination were carried out using the SHELXTL package.[S2] Relevant crystal data collection and refinement data for the crystal structures of **1**, **2** and **3** are summarized in Table S1. In the refinement procedure for **2**, the crown ring (O6-O9) is disordered over two sites occupied in a 62:38 ratio. The disorder components were found from difference electron density maps and refined with fractional occupancies (see Figure S4). In the lattice, one chloroform molecule is disordered over two positions with occupancies of 61:39 (see Figure S4).

Table S1 Crystallographic data and refinement parameters of **1-3**

	**1**	**2**	**3**
formula	$C_{54}H_{58}Cl_3O_{11}Rb$	$C_{55}H_{54}Cl_9CsO_{10}$	$C_{53}H_{55}Cl_3Cs_2O_{11}$
formula weight	1074.82	1325.93	1240.14
crystal system	173 (2)	173 (2)	173 (2)
space group	Triclinic	Monoclinic	Monoclinic
a (Å)	*P*-1	$P2_1/n$	$P2_1/c$
b (Å)	13.4400(3)	13.1773(15)	13.166(5)
c (Å)	14.3933(3)	20.344(2)	31.338(12)
α (deg)	14.8036(3)	22.581(3)	14.278(5)
β (deg)	84.0730(10)	90	90
γ (deg)	76.7280(10)	106.172(3)	117.28(2)
V (Å^3)	62.7770(10)	90	90
Z	2478.47(9)	5814.2(12)	5236(4)
D_{calc}(g/cm^3)	2	4	4
μ (mm^{-1})	1.440	1.515	1.573
$2\theta_{max}$ (deg)	1.222	1.106	1.604
reflections collected	26.00	28.31	28.00
independent reflections	57893	57934	73709
goodness-of-fit on F^2	9744 [R_{int} = 0.0412]	14416 [R_{int} = 0.0342]	12632 [R_{int} = 0.0590]
R_1, wR_2 [$I > 2\sigma(I)$]	1.044	1.030	1.031
R_1, wR_2 (all data)	0.0460, 0.1194	0.0462, 0.1205	0.0488, 0.1126

The CCDC reference numbers 2084082 (**1**), 874813 (**2**), and 874814 (**3**).

Table S2 Selected Bond Lengths (Å) and Bond Angles (deg) for **1**

Rb1-O1	3.0797(18)	Rb1-O9	3.143(2)
Rb1-O5	3.1052(17)	Rb1-O10	2.9114(17)
Rb1-O8	2.924(2)	Rb1-O11	2.879(3)
O1-Rb1-O5	89.32(5)	O5-Rb1-O8	120.53(6)
O1-Rb1-O8	113.26(6)	O5-Rb1-O9	88.80(5)
O1-Rb1-O9	165.29(5)	O5-Rb1-O10	68.63(5)
O1-Rb1-O10	136.87(5)	O5-Rb1-O11	154.20(7)
O1-Rb1-O11	64.88(7)	O9-Rb1-O11	116.01(7)
O8-Rb1- O9	56.00(6)	O9-Rb1-O10	55.00(5)
O8-Rb1- O10	109.87(6)	O10-Rb1-O11	130.58(8)
O8-Rb1-O11	73.01(7)		

Table S3 Selected Bond Lengths (Å) and Bond Angles (deg) for **2**

Cs1-O1	3.377(2)	Cs1-O2	3.621(2)
Cs1-O3	3.653(2)	Cs1-O4	3.187(2)
Cs1-O5	3.059(2)	Cs1-O6	3.308(6)
Cs1-O7	3.103(7)	Cs1-O8	3.193(11)
Cs1-O9	3.556(7)	Cs1-O10	3.056(2)
O1-Cs1-O2	41.50(5)	O2-Cs1-O3	38.51(5)
O1-Cs1-O3	74.21(7)	O2-Cs1-O4	78.34(8)
O1-Cs1-O4	98.95(6)	O2-Cs1-O5	114.47(6)
O1-Cs1-O5	72.98(6)	O2-Cs1-O6	110.47(11)
O1-Cs1-O6	86.23(12)	O2-Cs1-O7	81.23(17)
O1-Cs1-O7	92.48(17)	O2-Cs1-O8	107.69(3)
O1-Cs1-O8	144.5(2)	O2-Cs1-O9	159.52(3)
O1-Cs1-O9	156.29(14)	O2-Cs1-O10	146.39(5)
O1-Cs1-O10	120.92(6)	O3-Cs1-O4	43.44(5)
O4-Cs1-O5	121.73(6)	O3-Cs1-O5	139.91(6)
O4-Cs1-O6	170.49(12)	O3-Cs1-O6	146.05(11)
O4-Cs1-O7	133.39(15)	O3-Cs1-O7	98.94(16)
O4-Cs1-O8	87.48(15)	O3-Cs1-O8	87.75(15)
O4-Cs1-O9	98.97(13)	O3-Cs1-O9	129.37(13)
O4-Cs1-O10	77.84(6)	O3-Cs1-O10	121.14(5)
O5-Cs1-O6	52.07(12)	O6-Cs1-O7	53.72(19)
O5-Cs1-O7	104.85(15)	O6-Cs1-O8	92.9(2)
O5-Cs1-O8	132.13(17)	O6-Cs1-O9	74.03(14)
O5-Cs1-O9	84.41(14)	O6-Cs1-O10	92.65(12)
O5-Cs1-O10	61.11(6)	O7-Cs1-O8	59.8(2)
O8-Cs1-O9	51.8(2)	O7-Cs1-O9	86.4(2)
O8-Cs1-O10	94.6(2)	O7-Cs1-O10	132.30(18)
O9-Cs1-O10	49.03(13)		

Table S4 Selected Bond Lengths (Å) and Bond Angles (deg) for **3**

Cs1-O3	3.145(3)	Cs1-O9	3.110(4)
Cs1-O4	3.200(3)	Cs1-O10	3.173(3)
Cs1-O5	3.098(3)	Cs2-O1	3.046(3)
Cs1-O6	3.376(4)	Cs2-O4	3.063(3)
Cs1-O8	3.250(5)	Cs2-O1W	3.079(3)
O3-Cs1-O4	44.91(7)	O4-Cs1-O5	119.16(8)
O3-Cs1-O5	74.87(8)	O4-Cs1-O6	143.41(8)
O3-Cs1-O6	108.27(8)	O4-Cs1-O8	82.49(10)
O3-Cs1-O8	107.77(10)	O4-Cs1-O9	116.88(9)
O3-Cs1-O9	158.26(10)	O4-Cs1-O10	71.35(7)
O3-Cs1-O10	115.20(7)	O5-Cs1-O6	51.25(8)
O6-Cs1-O8	85.53(10)	O5-Cs1-O8	132.67(9)
O6-Cs1-O9	80.82(9)	O5-Cs1-O9	123.81(9)
O6-Cs1-O10	133.64(8)	O5-Cs1-O10	156.31(8)
O8-Cs1-O9	52.18(12)	O9-Cs1-O10	52.82(8)
O8-Cs1-O10	66.94(9)	O1-Cs2-O4	89.42(8)
O4-Cs2-O1W	73.93(9)	O1-Cs2-O1W	72.35(9)

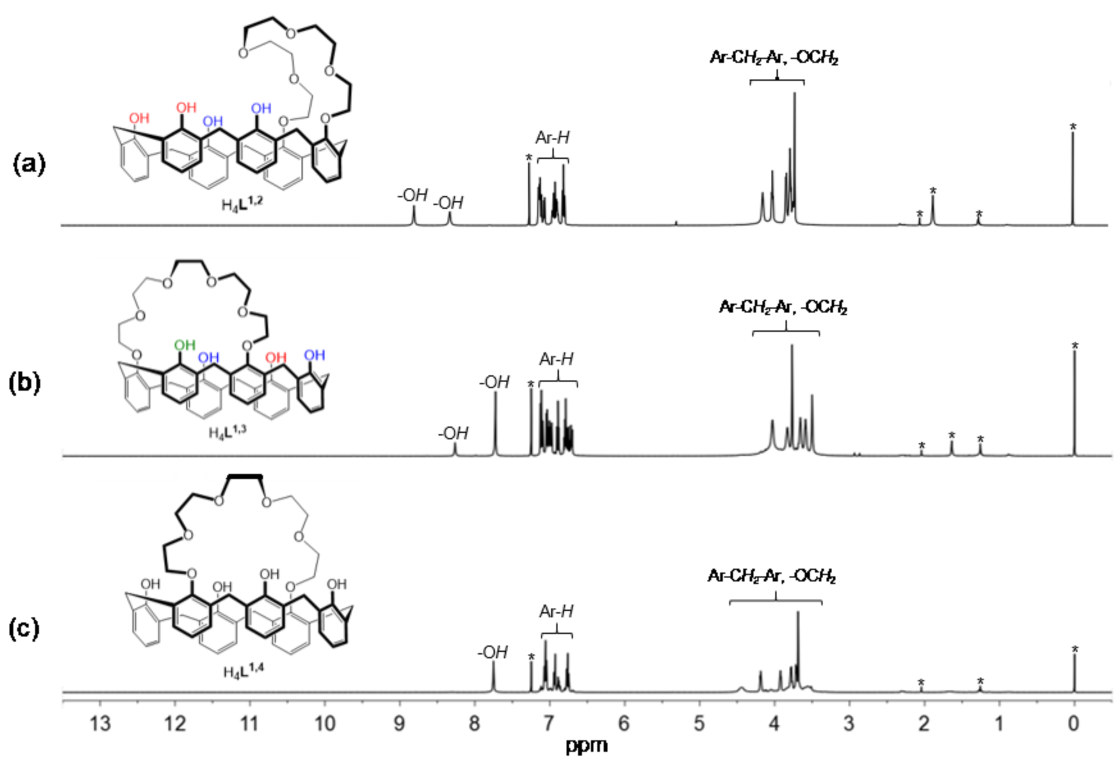

Figure S1 ^{1}H NMR (500 MHz) spectra of (a) $H_4L^{1,2}$, (b) $H_4L^{1,3}$, and (c) $H_4L^{1,4}$ in $CDCl_3$.

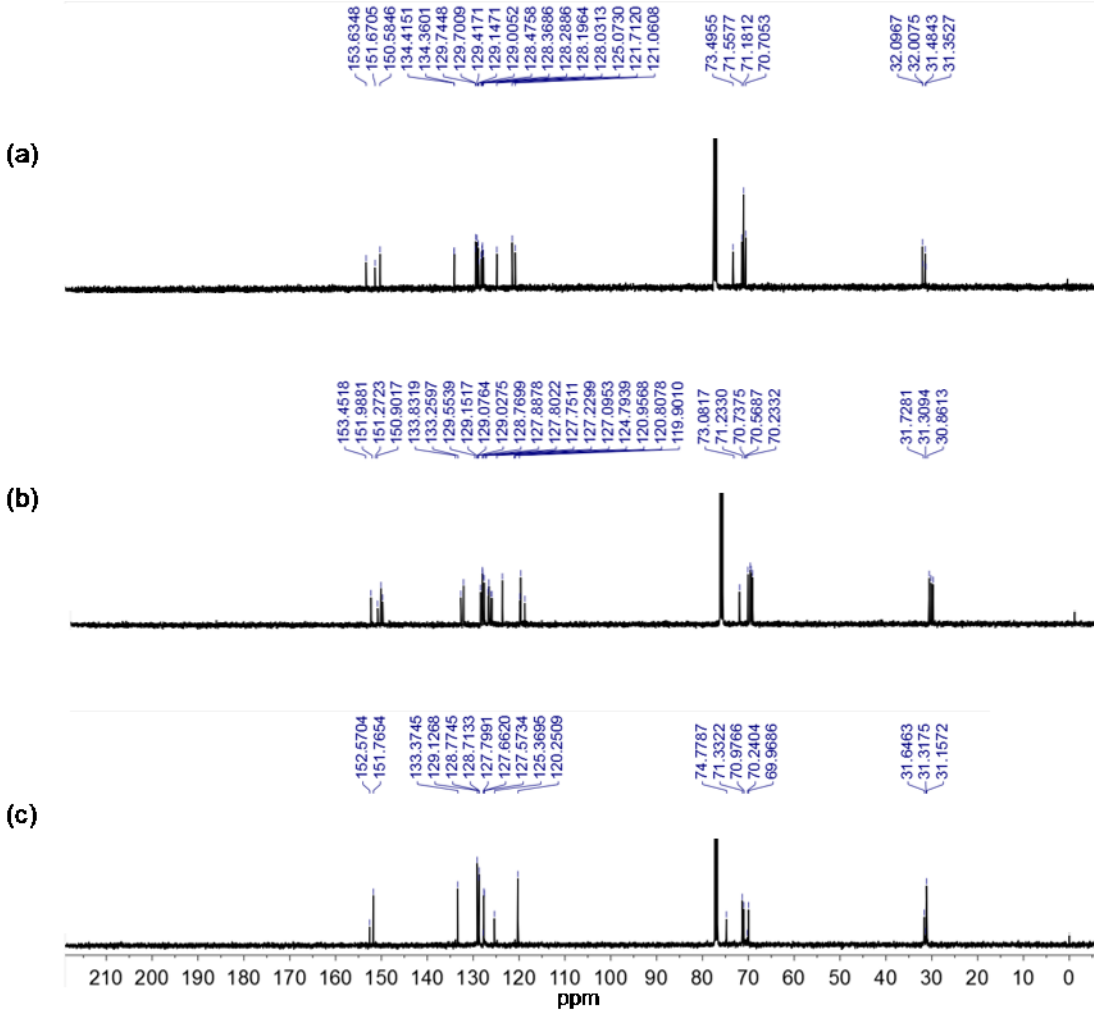

Figure S2 ^{13}C NMR (125 MHz) spectra of (a) $H_4L^{1,2}$, (b) $H_4L^{1,3}$, and (b) $H_4L^{1,4}$ in $CDCl_3$.

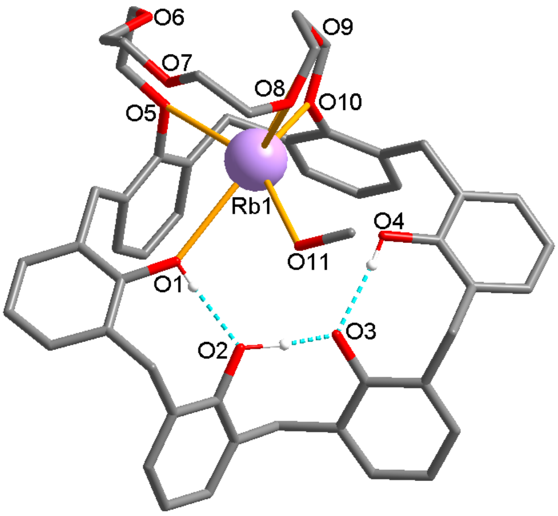

(a)

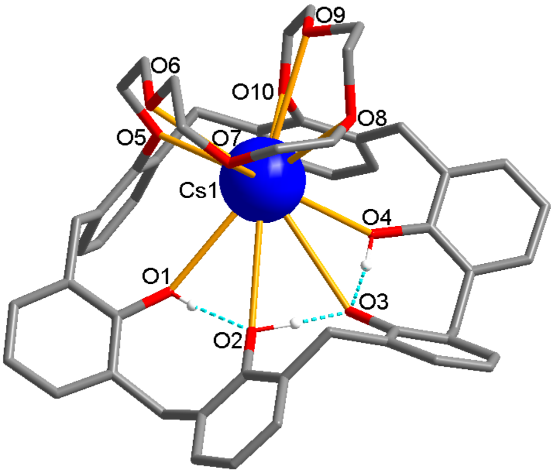

(b)

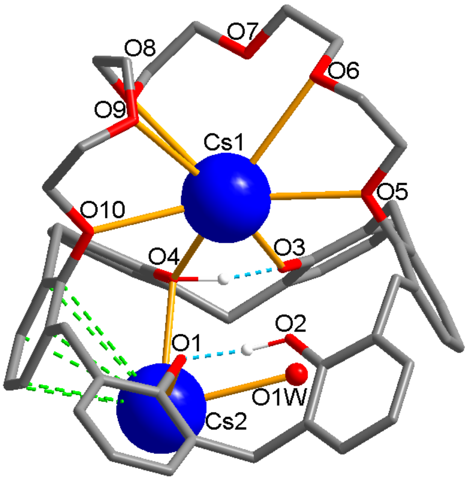

(c)

Figure S3 Crystal structures of (a) **1**, (b) **2**, and (c) **3**, showing the intramolecular H-bonds (light blue dashed lines) and cation-π interactions (green dashed lines).

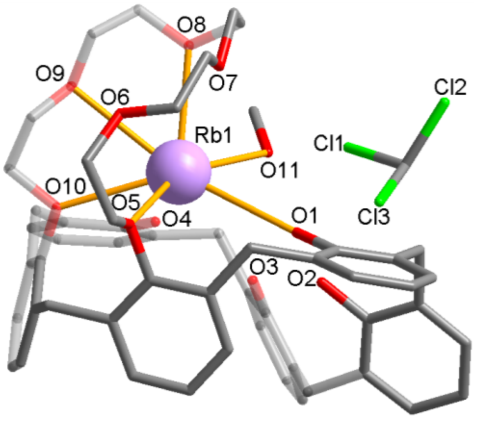

(a)

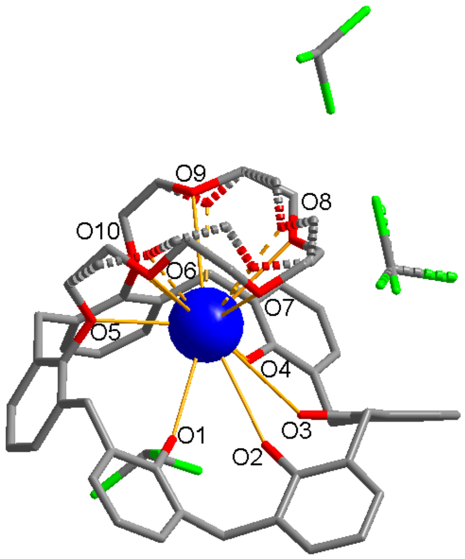

(b)

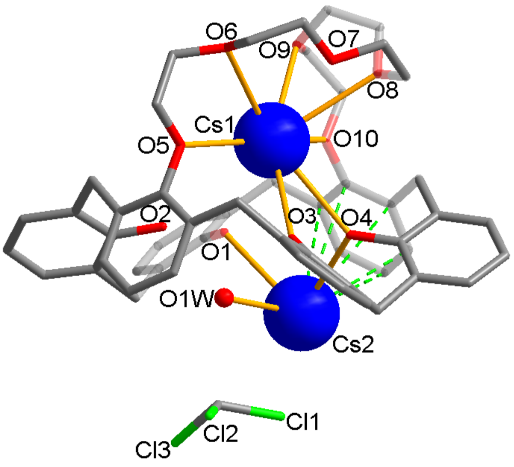

(c)

Figure S4 Crystal structures of (a) **1**, (b) **2**, and (c) **3**, showing the lattice solvent molecules. The crystal structure of **2** shows the disordered crown loop (62:38) and chloroform molecule (61:39).
